# Study on web pillar failure mechanism during auger mining and its associated risk assessment

**DOI:** 10.1038/s41598-023-34252-2

**Published:** 2023-05-04

**Authors:** Juyu Jiang, Jianxiong Sun, Dong Wang, Ye Lu, Mingxiang Cai, Lei Li

**Affiliations:** 1 College of Mining Liaoning Technical University, Fuxin, 123000 Liaoning China; 2 Guoneng Xinjiang Zhundong Energy Co., Ltd., Changji, 831100 Xinjiang China; 3 China Coal Technology and Industry Energy Technology Development Co., Ltd., Beijing, 100029 China

**Keywords:** Coal, Mineralogy

## Abstract

To safely and efficiently recover trapped coal under final endwalls in open cut mines, theoretical analysis and numerical calculation were used to study the stability of web pillar during auger mining. The partial ordered set (poset) evaluation model was used to develop a risk assessment methodology, and auger mining at Pingshuo Antaibao open cut coal mine was used as a field example for validation. Based on the catastrophe theory, the failure criterion of web pillar was established. From the limit equilibrium theory, the maximum allowable plastic yield zone width and minimum web pillar width were proposed under various Factor of Safety (FoS) thresholds. This in turn provides a new method for web pillar design. Based on the poset theory and combining with the risk evaluation and proposed hazard levels, input data were standardized and weighted. Subsequently, the comparison matrix, HASSE matrix and HASSE diagram were established. The study shows that: when the width of the plastic zone of web pillar exceeds 88% of the total width, web pillar may be unstable. Based on the calculation formula for the required width of web pillar, the required pillar width was 4.93 m and it was deemed as “mostly stable”. This was consistent with the field condition on site. Such that this method was validated.

## Introduction

China has a series of open cut coal mines, especially in Inner Mongolia and Shaanxi. However, due to geological or mining constraints, it is inevitable to leave some high quality coal under final endwalls. This will cause a huge waste of resources. Hence, auger (highwall) mining at final endwalls have received significant attention as it can recover additional coal for open cut operations. Due to rapid advancement of such technology, a number of equipment and methods have been introduced, including auger mining via continuous miner and blast and buttress method. To prevent overlying strata from collapsing and subsequent endwall stability, web pillars (coal pillars) are generally left between auger holes^1–4^. Therefore, it is particularly important to study the failure mechanisms of web pillar and provide adequate designs for auger mining. This is of great significance for ensuring the safety of equipment and personnel and preventing disasters such as endwall failure or nearby levee failure.

Numerous studies have been conducted to investigate the stability of web pillars under final endwalls during auger mining^5^. Outside China, pillar sizes are mainly governed based on strength empirical formulae^6,7^. Porarthur et al.^8^ used a hybrid method based experience and numerical simulation to design web and pillar in India; the study introduced a correction factor (constant) into the strength empirical equation when aspect ratio (width vs. height) is less than 1. This resulted in more practical parameters. Deliveris and Benardos^9^ used two-dimensional numerical simulation and three-dimensional mathematical analysis to study the approximate solution; and evaluated geomechanical responses of lignite pillars formed by the web and pillar. They pointed out that the 2D approximation method can be used for 3D problems. Lukáš et al.^10^ used anchor bolts to support overlying strata so that solve the problem of pillar stability and fully reinforced several existing pillars. Hikaru et al.^11^ considered recovery rate and the design of suitable web pillar width using numerical analysis. Zhang et al.^12^ used FLAC3D numerical simulation software to systematically investigate the coefficient of stress increase and stability of web pillar; they subsequently proposed a formula that describing the relationship between stress increase coefficient of upper and lower pillars and the interlayer properties. In China, Chen and Wu^13,14^ applied the cusp catastrophe theory and limit analysis method to deduce the instability criterion. Wang et al.^15–17^ used theoretical analysis, creep test, numerical simulation and field validation to study the stability of auger mining pillars under slopes. They subsequently proposed a parameter design method. Peng et al.^18,19^ developed a method for Factor of Safety (FoS) and minimum pillar width calculations.

The partially ordered set evaluation method provides high level of objectivity and is applicable to many research fields^20^. For instance, Halfon et al.^21^ used Hasse diagram to show the comparison of various chemical pollution levels based on chemical pollution evaluation standard. Badinger and Heinrich ^22^ used partially ordered set theory to study the financial systems of 81 countries and obtained evaluation criterion for financial system design. For a partially ordered set, if all possible linear extensions are deduced, then the average ranking of individual elements can be calculated based on it. Subsequently, the comparison of schemes can be completed^23^; Brüggemann et al.^24^ provided an effective approximation method through concept of upper set, lower set and interval set. Loof et al.^25^ further improved the technique proposed by Bruggemann et al.^24^ in the average height formula. The improved formula provided higher accuracy. To ensure the safe production at mining face, Wang et al.^26^ combined AHP and set pair analysis theories and constructed a model to analyse the definite and uncertain data. Eventually, the risk assessment of spontaneous combustion in goaf was proposed. Chen et al.^27^ applied partially ordered set theory to evaluate the probability of spontaneous combustion in goaf for the first time. Jia et al.^28^ was able to predict the coal spontaneous combustion in goaf areas based on such method. Given the partially ordered set theory has been approved reliable for risk assessment, it can be used to determine the stability of s under final endwalls.

Therefore, this study proposed a risk assessment table to standardise the pillar stability index and associated risk levels based on field data and partially ordered set. The proposed assessment is able to standardise input data and sort weighting on each parameter. Based on validation with field data, it is clear that such method provides a reasonable and feasible solution for stability during auger mining.

## Determination of catastrophic failure during auger mining

The main reason for failure and instability of supporting web pillar is that loading on the web pillar is greater than its ultimate strength, and plastic zones are formed on both sides of the web pillar. Under static loading, load on web pillar is the load of overlying strata^29^. According to theory of two-zone constraint proposed by Wilson^30^, the web pillar is divided into a plastic zone and an elastic core zone, in which the elastic zone is surrounded by the plastic zone and is constrained by the plastic zone.

**Figure 1:**
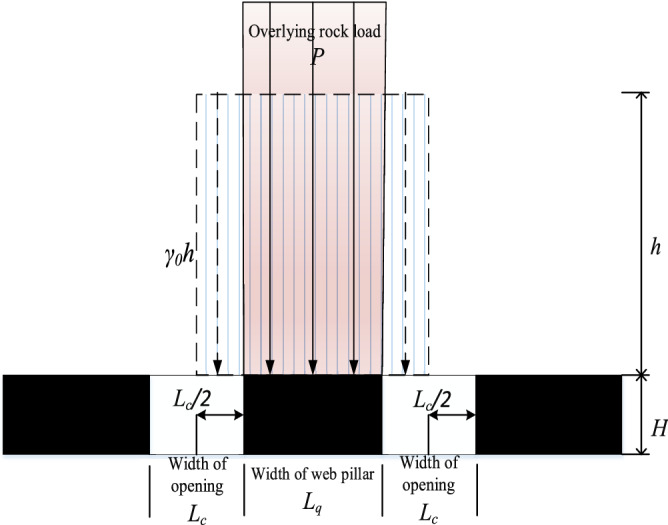
Mechanical model of highwall mining.

Assuming width of auger opening is *L*_*c*_, width of web pillar is *L*_*q*_, *h* is the average thickness of overlying strata and bulk density is *γ*_*0*_. Loading of the overlying strata on web pillar is illustrated in Fig. 1. Based on the efficient area theory^8^, loading on web pillar can be expressed as:

1 $$P = \gamma_{0} h(L_{c} + L_{q} )/L_{q}$$

Vertical stress acting on the web pillar induced by auger mining is concentrated on both sides of the pillar, whereas vertical stress is less in the middle. When stress exceeds the strength of coal body, a plastic zone is formed on pillar side. It then gradually develops from the two sides to the pillar core^31^ and eventually leads to pillar failure. This failure process is typically non-linear and has a trend that can be interpreted by cusp catastrophe theoretical analysis^32,33^.

**Figure 2:**
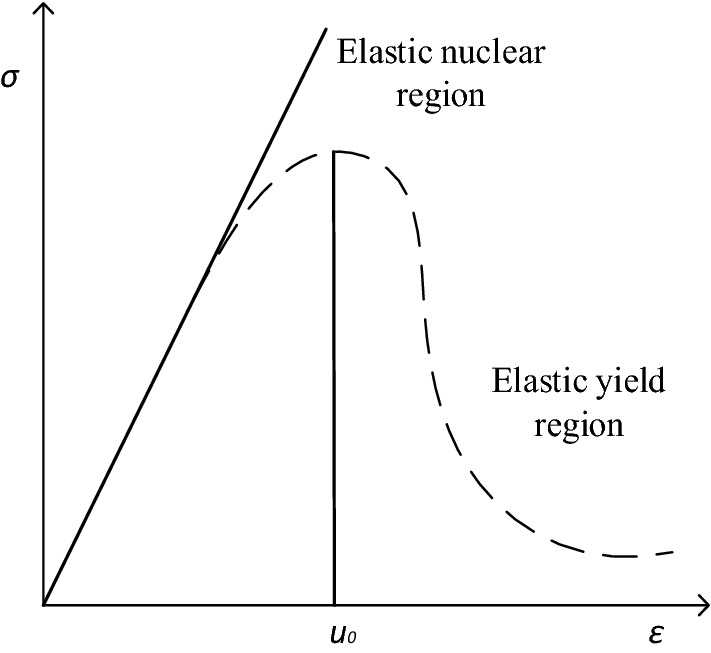
Stress–strain curve of a web pillar.

Auger mining leads to redistribution of stress. Stress in the roof above auger opening is distributed to its adjacent coal pillars; such that symmetrical plastic zones are formed on both sides of pillars. On the other hand, core pillar is still under elastic condition and behaves differently than either side. As shown in Fig. 2, stress and strain curve in plastic zone exhibits a softening nonlinear relationship’ in which its ability to resist deformation decreases with the increase of the strain value, until it reaches the peak strength and then rapidly drops post failure. On the other hand, in the elastic core area, the stress and strain curve is linearly correlated. In this zone, the strength of pillar high and its ability to resist deformation increases with the increase of the strain value.

At the same time, *H* is thickness coal seam (m); ε is strain of under a certain load (%); *E* is elastic modulus of pillar (MPa); *u* is deformation of pillar (m); *u*_0_ is compression under a certain load (m).

The loading (Ps) under corresponding *u* can be expressed as:

2 $$P_{s} = \frac{{2L_{q} Eu}}{H}e^{{\left( { - \frac{u}{{u_{0} }}} \right)}}$$

The elastic loading (Ps) under corresponding *u* can be expressed as:

3 $$P_{v} = \frac{Eu}{H}\left( {L_{q} - 2X_{q} } \right)$$

Hence, total potential energy of the system (V) can be expressed as:

4 $$V = \int_{0}^{u} {P_{s} du + } \int_{0}^{u} {P_{e} du - \gamma_{0} h(L_{c} + L_{q} )u}$$

By differentiate the function by u, the derivative can be calculated as:

5 $$V^{\prime} = P_{s} + P_{v} - \gamma_{0} h(L_{c} + L_{q} )$$

Based on the smoothness of equilibrium curve, a cusp can be obtained:

6 $$V^{\prime\prime\prime} = \frac{{P_{s} }}{{Hu_{0} }}\left(\frac{u}{{u_{0} }} - 2\right)$$

Assuming that total width of the plastic zones on both sides is 2*X*_*q*_ and width of the elastic core zone is *L*_*q *_− 2*X*_*q*_. When *u* = 2*u*_0_, the cusp is located at the inflection point of curve. To establish the cusp catastrophe model, the equilibrium curve function is expanded at *u* = 2*u*_0_. Based on Taylor’s formula only taking the first three items, it can be expressed as:

7 $$\frac{{8X_{q} Eu_{0} e^{{ - 2}} }}{{3h}}\left\{ {\left( {\frac{{u - 2u_{0} }}{{2u_{0} }}} \right)^{3} + \frac{3}{2}\left[ {\frac{{\left( {L_{q} - 2X_{q} } \right)e^{2} }}{{2X_{q} }} - 1} \right]*\frac{{u - 2u_{0} }}{{2u_{0} }} + \frac{3}{2}\left[ {1 + \frac{{\left( {L_{q} - 2X_{q} } \right)e^{2} }}{{2X_{q} }} - \frac{{PHe^{2} }}{{4X_{q} Eu_{0} }}} \right]} \right\} = 0$$

By introducing the dimensionless constant *z* as the state variable; *p* and *q* as the control variable where k_0_ and t are the intermediate variables:

8 $$\left\{ {\begin{array}{*{20}l} {z = \frac{{u - 2u_{0} }}{{2u_{0} }},p = \frac{3}{2}\left( {1 + k_{0} - t} \right)} \hfill \\ {k_{0} = \frac{{\left( {L_{q} - 2X_{q} } \right)e^{2} }}{{2X_{q} }},t = \frac{{\gamma hHe^{2} }}{{4X_{q} Eu_{0} }}\left( {L_{c} + L_{q} } \right)} \hfill \\ \end{array} } \right.$$

The balanced equation of cusp can hence be deduced as:

9 $$z^{3} + pz + q = 0$$

By combining Eq. (9) with inflection point Eq. 3*z*^2^ + *p* = 0, a bifurcation function can be obtained:

10 $$\Delta = 2\left( {k_{0} - 1} \right)^{3} + 9\left( {1 + k_{0} - t} \right)^{2} = 0$$

By substituting dimensionless constants from Eq. (8) to Eq. (10):

11 $$\Delta = 2\left[ {\frac{{\left( {L_{q} - 2X_{q} } \right)e^{2} }}{{2X_{q} }} - 1} \right]^{3} + 9\left[ {1 + \frac{{\left( {L_{q} - 2X_{q} } \right)e^{2} }}{{2X_{q} }} - \frac{{he^{2} }}{{4X_{q} Eu_{0} }}PL_{q} } \right]^{2} = 0$$

When Δ = 0, web pillar is under the critical state of system instability. When Δ < 0, the system may cross the bifurcation set and undergo sudden changes to become unstable. The necessary condition for the sudden instability is (*L*_*q *_*− *2*X*_*q*_)*e^2^/2*X*_*q *_− 1 < 0, in which the solution is 2*X*_*q*_ > 0.88*L*_*q*_. In other words, when width of the pillar plastic zone accounts for more than 88% of the total width, web pillar may be suddenly unstable.

## Constitution model of minimum pillar width

Based on Mohr–Coulomb failure criterion, failure of rock mass is caused by shear stress acting on rock mass exceeding its shear strength. The shear stress is closely related to the properties of material and the friction on failure surface^31^. In auger mining, the Mohr strength envelope can be replaced by a straight line, that is, the Coulomb formula *τ* = *c* + *σtanφ*. Its basic expression can be transformed into:

12 $$\sigma_{1} = \frac{1 + \sin \varphi }{{1 - \sin \varphi }}\sigma_{3} + \frac{2c\cos \varphi }{{1 - \sin \varphi }}$$

in which σ_1_ is the maximum principal stress (MPa); σ_3_ is the minimum principal stress (MPa); c is the cohesion of the coal body (MPa); φ is the internal friction angle of the coal body (°).

Once auger mining is completed, both sides of coal pillar are goaf and the coal body must be compressed by the roof and floor strata to both sides. Shear stress is generated at the contact surfaces between the coal pillar and the roof. The maximum principal stress σ_1_ is the vertical stress acting on coal pillar, and the minimum principal stress σ_3_ is the horizontal stress on coal pillar. Assuming σ_0_ is the vertical stress mining, the magnitude can be expressed as *γ*_*0*_*h*. Hence, the horizontal stress is *λγ*_*0*_*h* and *λ* is the lateral pressure coefficient of the coal seam.

**Figure 3:**
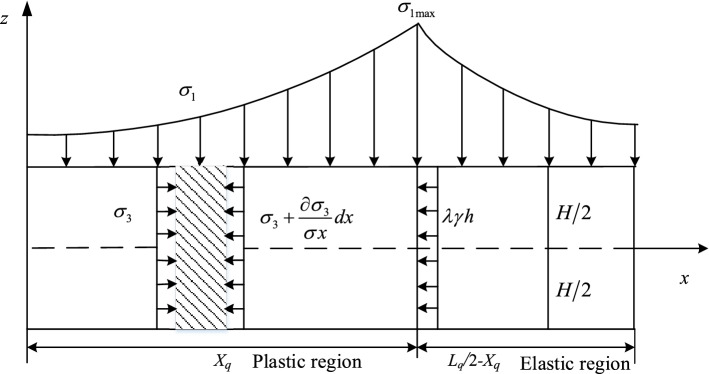
Mechanical analysis model of the web pillar.

As the stress distribution of opening and coal pillar are symmetrical, a mechanical analysis model on one side of the coal pillar was established (Fig. 3). Horizontal stress of the coal pillar gradually increases from outside to inside and reaches its peak at the transition between plastic zone and elastic zone^34^. At this point, the horizontal stress magnitude is *λγ*_*0*_*h* and the corresponding maximum vertical stress *σ*_1max_ of the coal pillar can be expressed as:

13 $$\sigma_{1\max } = \frac{1 + \sin \varphi }{{1 - \sin \varphi }}\lambda \gamma_{0} h + \frac{2c\cos \varphi }{{1 - \sin \varphi }}$$

**Figure 4:**
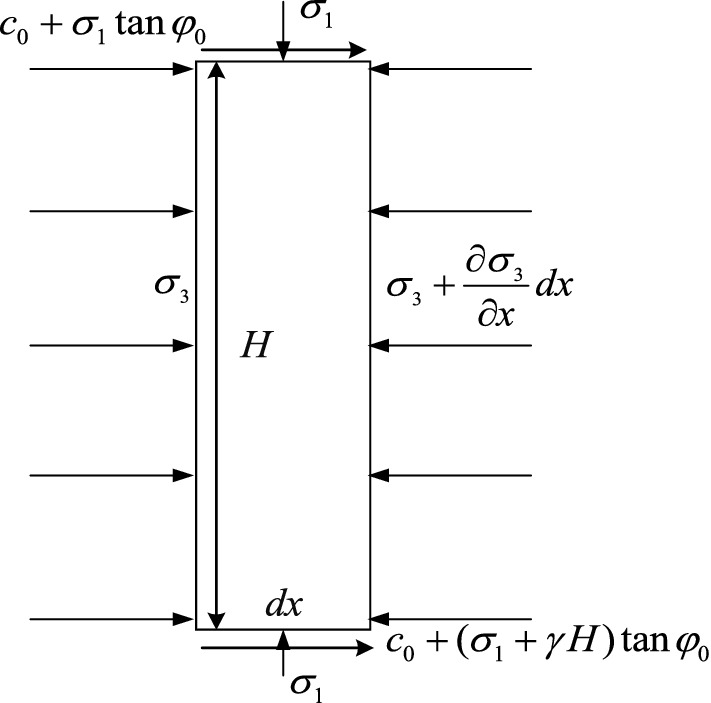
Micro mechanical model of the web pillar.

Take a point (Fig. 4) in the x-axis direction of the web pillar model and establish the force balance equation in the x-axis direction:

14 $$H\sigma_{{3}} - H\left( {\sigma_{{3}} + \frac{{\partial \sigma_{{3}} }}{\partial x}dx} \right) + \left[ {2c_{{0}} + \left( {2\sigma_{{1}} + \gamma H} \right)\tan \varphi_{{0}} } \right] = 0$$

In which *c*_0、_*φ*_0_ are cohesion and friction angles of contact surfaces between pillar and roof and floor. *γ* is the bulk density of coal seam (MN/m^3^).

By simplifying Eq. (14):

15 $$\frac{{\partial \sigma_{3} }}{\partial x} = \frac{2}{H}\left( {c_{0} + \sigma_{1} \tan \varphi_{0} } \right) + \gamma \tan \varphi_{0}$$

By substituting Eq. (12) to Eq. (15), the derivative of* σ*_3_ can be obtained:

16 $$\frac{{\partial \sigma_{3} }}{\partial x} = \frac{2}{H}\left( {c_{0} + \left( {\frac{1 + \sin \varphi }{{1 - \sin \varphi }}\sigma_{3} + \frac{2c\cos \varphi }{{1 - \sin \varphi }}} \right)\tan \varphi_{0} } \right) + \gamma \tan \varphi_{0}$$

By substituting the boundary condition: x = 0 and horizontal stress *σ*_3_ = 0 at boundary of the coal pillar into Eq. (16), horizontal and vertical stresses in the plastic zone of the coal pillar can be calculated:

17 $$\left\{ {\begin{array}{*{20}l} {\sigma _{3}^{p} = \left( {\frac{{2c\cos \varphi }}{{1 + \sin \varphi }} + \frac{{c_{0} \left( {1 - \sin \varphi } \right)}}{{\tan \varphi _{0} \left( {1 + \sin \varphi } \right)}} + \frac{{\gamma H\left( {1 - \sin \varphi } \right)}}{{2\left( {1 + \sin \varphi } \right)}}} \right)e^{{\frac{{2\tan \varphi \left( {1 + \sin \varphi } \right)}}{{H\left( {1 - \sin \varphi } \right)}}x}} } \hfill \\ \quad { - \left( {\frac{{2c\cos \varphi }}{{1 + \sin \varphi }} + \frac{{c_{0} \left( {1 - \sin \varphi } \right)}}{{\tan \varphi _{0} \left( {1 + \sin \varphi } \right)}} + \frac{{\gamma H\left( {1 - \sin \varphi } \right)}}{{2\left( {1 + \sin \varphi } \right)}}} \right)\quad \quad \quad 0 \le x \le X_{q} } \hfill \\ {\sigma _{1}^{p} = \left( {\frac{{2c\cos \varphi }}{{1 + \sin \varphi }} + \frac{{c_{0} }}{{\tan \varphi _{0} }} + \frac{{\gamma H}}{2}} \right)e^{{\frac{{2\tan \varphi \left( {1 + \sin \varphi } \right)}}{{H\left( {1 - \sin \varphi } \right)}}x}} - \left( {\frac{{c_{0} }}{{\tan \varphi _{0} }} + \frac{{\gamma H}}{2}} \right)} \hfill \\ \end{array} } \right.$$

According to stress distribution and boundary conditions in the elastic core zone^35^, the vertical stress changes along the x-axis direction is:

18 $$\sigma_{1}^{e} = \sigma_{0} \left[ {1 - \left( {\frac{{X_{q} }}{x}} \right)^{2} } \right] + \sigma_{{1\left( {x = X_{q} } \right)}}^{p} \left( {\left( {\frac{{X_{q} }}{x}} \right)^{2} } \right)\begin{array}{*{20}c} {} & {} \\ \end{array} \left( {X_{q} \le x \le \frac{{L_{q} }}{2}} \right)$$

Stress acting on the coal pillar is redistributed post auger mining and the induced loading is equally shared by the coal pillars on both sides:

19 $$\frac{{1}}{{2}}L_{c} \sigma_{0} = \int_{0}^{{X_{q} }} {\left( {\sigma_{1}^{p} - \sigma_{0} } \right)} dx + \int_{{X_{q} }}^{{{{L_{q} } \mathord{\left/ {\vphantom {{L_{q} } 2}} \right. \kern-0pt} 2}}} {\left( {\sigma_{1}^{e} - \sigma_{0} } \right)} dx$$

By solving simultaneous equations and let *Y* = 2(1 + sin*φ*)*X*_*q*_tan*φ*_0_/(1 − sin*φ*)*H*, Eq. (19) can be simplified as:

20 $$\begin{aligned} \frac{{L_{c} \gamma _{0} h\left( {1 + \sin \varphi } \right)\tan \varphi }}{{\left( {1 - \sin \varphi } \right)H}} = & \left( {\frac{{2c\cos \varphi }}{{1 - \sin \varphi }} + \frac{{c_{0} }}{{\tan \varphi _{0} }} + \frac{{\gamma H}}{2}} \right)\left( {e^{Y} + Ye^{y} - \frac{{2X_{q} }}{{L_{q} }}Ye^{Y} - 1} \right) \\ & - \left( {\gamma _{0} h - \frac{{\gamma _{0} hX_{q} }}{{L_{q} }} + \frac{{c_{0} }}{{\tan \varphi _{0} }} + \frac{{\gamma H}}{2}} \right)Y + \left( {\frac{{c_{0} }}{{\tan \varphi _{0} }} + \frac{{\gamma H}}{2}} \right)\frac{{2X_{q} }}{{L_{q} }}Y \\ \end{aligned}$$

Based on aforementioned critical conditions for sudden instability of coal pillars, when width of the plastic zone on both sides of the coal pillar is 0.88 times of the coal pillar width, that is, 2*X*_*q*_ > 0.88*L*_*q*_, the web pillar is in a critical instability state. By substituting *L*_*q*_ = 2*X*_*q*_/0.88 into Eq. (20) and combining with Eq. (17), the width of plastic zone at the critical instability state of the coal pillar can be determined. Subsequently, the maximum vertical stress on coal pillar can be calculated, which is equal to the ultimate strength *σ*_*zl*_ of web pillar:

21 $$\begin{aligned} \sigma _{{zl}} = & \left[ {1.12\gamma _{0} h + \frac{{c_{0} }}{{\tan \varphi _{0} }} + \frac{{\gamma H}}{2} - 0.12\sigma _{{zl}} } \right]\ln \left( {1 + \frac{{2\tan \varphi _{0} \left( {\sigma _{{zl}} - \sigma _{{zl}} \sin \varphi - 2\cos \varphi } \right)}}{{4c\cos \varphi \tan \varphi _{0} + 2c_{0} \left( {1 - \sin \varphi } \right) + \gamma H\tan \varphi _{0} \left( {1 - \sin \varphi } \right)}}} \right) \\ & + \frac{{L_{c} \gamma _{0} h\left( {1 + \sin \varphi } \right)\tan \varphi - 2Hc\cos \varphi }}{{\left( {1 - \sin \varphi } \right)H}} \\ \end{aligned}$$

Based on the strength theory, the FoS of coal pillar (*f*_*s*_) under the condition of non-uniform load during auger mining can be expressed as the ratio of its ultimate strength *σ*_*zl*_ to the applied stress:

22 $$f_{s} = \frac{{\sigma_{zl} }}{{\sigma_{{{\text{1max}}}} }}$$

By substituting Eq. (21) and Eq. (22) into Eq. (17), expression of the maximum allowable width of plastic zone of the coal pillar under different FoS can be obtained:

23 $$X_{q} = \frac{{H\left( {1 - \sin \varphi } \right)}}{{2\left( {1 + \sin \varphi } \right)\tan \varphi }}\ln \left[ {1 + \left( {\frac{{2\tan \varphi _{0} \left( {\sigma _{{zl}} - \sigma _{{zl}} \sin \varphi - 2cf_{s} \cos \varphi } \right)}}{{4cf_{s} \cos \varphi \tan \varphi + 2c_{0} f_{s} \left( {1 - \sin \varphi } \right) + \gamma Hf_{s} \tan \varphi _{0} \left( {1 - \sin \varphi } \right)}}} \right)} \right]$$

Based on the field experience of auger mining projects in the past, the FoS of web pillars is generally required to be above 1.3^8^. By substituting Eq. (23) into Eq. (20), the calculation formula for the required width of web pillar under different FoS is obtained:

24 $$L_{q} = \frac{{e^{Y} Y^{2} A}}{{Ie^{Y} + SYe^{Y} - G}}$$

$${\text{where}}\;\left\{ {\begin{array}{*{20}l} {A = \frac{{\left( {\sigma _{{{\text{zl}}}} - f_{s} \gamma _{0} h} \right)H\left( {1 - \sin \varphi } \right)^{2} }}{{\left( {1 + \sin \varphi } \right)\tan \varphi }}} \hfill \\ {I = \sigma _{{{\text{zl}}}} \left( {1 - \sin \varphi } \right) + \frac{{\left( {2f_{s} c_{0} + f_{s} \gamma H\tan \varphi _{0} } \right)\left( {1 - \sin \varphi } \right)}}{{2\tan \varphi _{0} }} - \frac{{L_{c} f_{s} \left( {1 + \sin \varphi } \right)\tan \varphi _{0} \gamma _{0} h}}{H}} \hfill \\ {S = \sigma _{{{\text{zl}}}} \left( {1 - \sin \varphi } \right) - \frac{{\left( {2f_{s} c_{0} + f_{s} \gamma H\tan \varphi _{0} } \right)\left( {1 - \sin \varphi } \right)}}{{2\tan \varphi _{0} }} - 2f_{s} \left( {1 - \sin \varphi } \right)\gamma _{0} h} \hfill \\ \begin{gathered} G = \sigma _{{{\text{zl}}}} \left( {1 - \sin \varphi } \right) + \frac{{\left( {2f_{s} c_{0} + f_{s} \gamma H\tan \varphi _{0} } \right)\left( {1 - \sin \varphi } \right)}}{{2\tan \varphi _{0} }} \hfill \\ Y = {\text{ln}}\left[ {{\text{1}} + \frac{{2\tan \varphi \left( {\sigma _{{{\text{zl}}}} - \sigma _{{{\text{zl}}}} \sin \varphi - 2cf_{s} \cos \varphi } \right)}}{{2cf_{s} \left( {1 + \sin \varphi } \right) + \gamma Hf_{s} \tan \varphi \left( {1 - \sin \varphi } \right)}}} \right] \hfill \\ \end{gathered} \hfill \\ \end{array} } \right.$$

## An evaluation of pillar instability under auger mining and its associated risk using partially ordered set

### Fundamentals of partially ordered set

A poset (A, ≤) means that the set A is equipped with a partial order ≤ , where the partial order ≤ is a binary relation on a non-empty set A that satisfies reflexivity, antisymmetry,and transitivity. When using this binary relationship to make problem decisions, it should be noted that the evaluation value and the evaluation relationship are not the same in essence. Yue et al.^36^ proposed a relationship between the two.

For ∀*x*,*y*∈*A*,

25 $$x \le y \Leftrightarrow c_{j} \left( x \right) \le c_{j} \left( y \right)\begin{array}{*{20}c} {} & {} & {} \\ \end{array} j = 1,2, \cdots ,n$$

where x and y are evaluation objects, *c*_*j*_ represents the jth evaluation criterion, *c*_*j*_(*x*) is the normalised value of x on the criterion cj, and *c*_*j*_(*y*) is the normalized value of y on the criterion *c*_*j*_.

Given the index weight satisfies *ω*_11_ > *ω*_12_ > ··· > *ω*_1n_, Chen et al.^27^ applied the matrix form to realise the implicit weighting of multi-sample multi-indicator decision-making problems.

26 $$D = \left( {d_{ij} } \right)_{m \times n} = XE = \left( {\begin{array}{*{20}c} {x_{11} } & {x_{11} + x_{12} } & \cdots & {x_{11} + x_{12} + \cdots + x_{1n} } \\ {x_{21} } & {x_{21} + x_{22} } & \cdots & {x_{21} + x_{22} + \cdots + x_{2n} } \\ \vdots & \vdots & \ddots & \vdots \\ {x_{m1} } & {x_{m1} + x_{m2} } & \cdots & {x_{m1} + x_{m2} + \cdots + x_{mn} } \\ \end{array} } \right)$$

In the equation: *X* is evaluation matrix, $$E = \left( {\begin{array}{*{20}c} 1 & 1 & \cdots & 1 \\ 0 & 1 & \cdots & 1 \\ \vdots & \vdots & \ddots & \vdots \\ 0 & 0 & \cdots & 1 \\ \end{array} } \right).$$

In the matrix D, if each value in the *m *− 1th row is greater than or equal to the value at the corresponding position in the m-th row, then the *m *− 1th evaluation object is better than or equal to the m-th evaluation object. For a given poset (A, ≤), ∀x,y ∈ *A*, if *x* is better than or equal to *y*, then *s*_*xy*_ = 1; otherwise *s*_*xy*_ = 0. *S* = (*s*_*xy*_)_*m*×*m*_ is hence a comparison matrix of (A, ≤).

The comparison matrix cannot provide direct visualisation of the partial order relationship, hence it is necessary to draw a partial order relationship diagram. The HASSE matrix can be obtained by transforming and calculating the comparison matrix, and the HASSE diagram can be drawn from the HASSE matrix. The HASSE graph is a special directed graph, which can visually present the partial order results of the evaluation objects in the form of a graph. It is a powerful tool to show the transfer relationship and structural relationship between evaluation objects. Fan^37^ developed a conversion relationship between the comparison matrix *S* and the HASSE matrix:

27 $$H_{s} = \left( {S - I} \right) - \left( {S - I} \right)^{2}$$

where *S* is a comparison matrix, *H*_*s*_ is a HASSE matrix, *I* is an identity matrix, and (*S *− *I*)^*2*^ represents a Boolean operation (i.e. 1 + 1 = 1, 1 + 0 = 1, 0 + 0 = 0, 1 × 1 = 1, 1 × 0 = 0, 0 × 0 = 0).

For any poset (A, ≤), the set *F*(*x*) = {*y* ∈ *A*|*x* ≤ *y*}} is the upper bound of A, and the set *O*(*x*) = {*y*∈*A*|*x* ≥ *y*} is the lower bound of A. The set *U*(*x*) is an incomparable set of A, where for any *x*∈*A*, the height of the scheme on the poset set (A, ≤)^38^ is

28 $$U\left( x \right) = A - O\left( x \right) - F\left( x \right)hav\left( x \right) = \frac{{\left| {F\left( x \right)} \right|}}{{\left| {F\left( x \right)} \right| + \left| {O\left( x \right)} \right|}}\left| {O\left( x \right)} \right| + \frac{{\left| {O\left( x \right)} \right|}}{{\left| {F\left( x \right)} \right| + \left| {O\left( x \right)} \right|}}\left( {\left| {O\left( x \right)} \right| + \left| {U\left( x \right)} \right|} \right)$$

where *|F*(*x*)*|, |O*(*x*)*|* and *|U*(*x*)*|* represent the number of elements in the sets *F*(*x*), *O*(*x*) and *U*(*x*) respectively. *Hav*(*x*) was then used to sort the entire set of solutions.

### Risk Assessment Model Construction and Implementation

The essential process of establishing a partially ordered set evaluation model is: Firstly, each index and grade interval of the sample to be evaluated are determined. Then, use the left and right end points of these grades as index values to divide into groups respectively. Based on the samples to be evaluated and the samples in each group, the risk level was subsequently obtained^39^. If a sample to be evaluated is between the samples constructed from the left and end points of the grade, then the sample to be evaluated must belong to this risk level.

Based on the field data in conjunction with on-site experience and relevant studies, a standardised risk assessment and weight ranking of the selected parameters of coal pillar instability were developed. The standard is constructed to mix graded samples into the sample group and use each sample in the overall ranking to reflect the degree of risk of the samples. The evaluation indicators were sorted according to the importance of their weights and the each indictor was ranked in order. The processed indicator data was accumulated column by column to obtain new indicator data. This accumulation process was called the process of implicit weighting.

**Figure 5:**
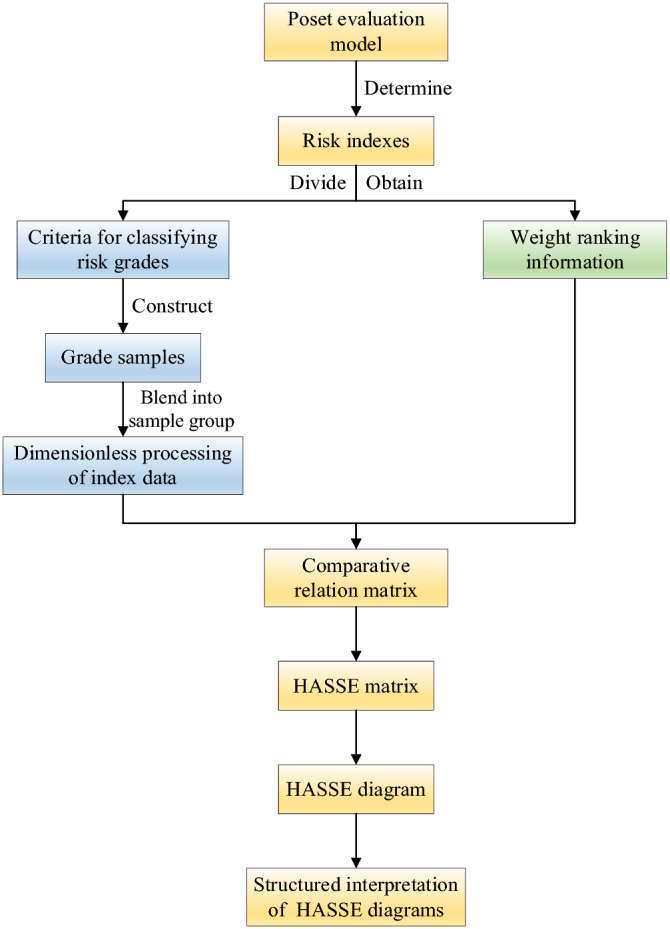
Flow chart of poset evaluation model.

By then, each indicator was compared and a comparison matrix was calculated, and the HASSE matrix was converted by Eq. (27). The HASSE diagram was subsequently drawn using the HASSE matrix and the clusters within the sample database was intuitively presented in the form of the diagram. The clusters were then used in Eq. (28) to calculate the height, which can in turn clarify the superiority and inferiority relationship within the layer set, and perform a structural interpretation of the HASSE diagram. Finally, the risk level of the sample was determined^40^. The process of poset evaluation model is shown in Fig. 5.

### Indicators on pillar web failure evaluation and associated hazard level

The evaluation indicators of web pillar instability risk are not unique and there are other factors that can influence the evaluation process, including mining parameters and mechanical properties of strata. The evaluation indicator is changing with the situations, weight of each indicators and associated the risk levels are therefore different^41^. Based on the geological, hydrogeological and rock mechnical information at Pingshuo Antaibao open cut coal mine, three hazard levels of web pillar instability were determined, namely stable, mostly stable and unstable, see Table 1 below. The indicators used in this study are depth of cover, auger opening width, pillar width, pillar strength and height.

**Table 1:** Classification standard of hazard level.

Evaluation indexes	Instability risk grade of end-slope mining support		
	Stable	Mostly stable	Unstable
Depth of cover /m	[50–80]	(80–160]	(160–200]
Width of opening /m	[1.2–1.8]	(1.8–3.5]	(3.5–5.0]
Width of web pillar /m	[6.0–4.0]	(4.0–2.5]	(2.5–1.0]
Ultimate strength /MPa	[12–8.5]	(8.5–4.5]	(4.5–1.5]
Height of web pillar /m	[1.8–3.5]	(3.5–5.0]	(5.0–6.5]

### Standardisation of evaluation index and weight ranking

There is often a dimensional relationship between different indicators. To eliminate the dimensional relationship between variables and ensure the data are comparable, it is essential to normalise the data. Normalization processing includes two types of processing methods: benefit type and cost type, i.e.:

29 $$y_{ij} = \frac{{x_{ij} - x_{\min } }}{{x_{\max } - x_{\min } }}$$

30 $$y_{ij} = \frac{{x_{\max } - x_{ij} }}{{x_{\max } - x_{\min } }}$$

The type of normalisation is generally determined on trends of indicators’ impacts on the results. For risk of web pillar instability, if the larger the indcator value the more stable the pillar is, then the benefit formula is used to for normlisation; otherwise the cost formula is used. In this case, the coal pillar width and ultimate strength should be normalised by the benefit formula Eq. (29), whereas the other indicators should be normliased by the cost formula Eq. (30).

Index weights are critical to any evaluation model involving weights. The poset evaluation model does not need to calculate accurate weights and the accurate prediction of samples can be realised by the weight sorting results^27^. The weight ranking in this study was derived from the field and the study results of Yin et al.^42^. The weighting of indicators in descending order is: web pillar width, depth of cover, coal pillar ultimate strength, opening width and coal pillar height, as shown in Table 2.

**Table 2:** Weight ranking.

Evaluation indexes	Depth of cover	Opening width	Width of pillar	Ultimate strength	Height of pillar
Weight order	2	4	1	3	5

## Field implementation

The EML340 type auger mining machine was used to recover the retained coal in the No. 4 coal seam of the south side of Antaibao open cut mine in Pingshuo. Height and width of the seam were 5 m and 3.3 m. The maximum depth of cover was 98.6 m. The physical and mechanical parameters of the nearby strata are shown in Table 3.

**Table 3:** Physical and mechanical parameters of coal and rock.

Lithology	Average thickness of stratum/m	The thickness of the rock at the deepest chamber/m	Unit weight *γ/*kN/m^3^	Modulus of Elasticity *E/*MPa	Poisson ratio *μ*	Cohesive */*MPa	Internal friction angle/°
Loess	36	0	19.5	8.6	0.31	0.028	22
Mudstone	133	98.6	24.6	1500	0.28	0.87	33
No.4 coal	11.9	11.9	14.1	291.67	0.31	0.21	36.8
Sandstone	36.5	36.5	24.6	2850	0.28	0.87	33

### Pillar ultimate strength and parameter design

Cohesion and internal friction angle of the interface between web pillar and roof /floor were taken as the coal cohesion and internal friction angle; and substituted into Eq. (19) to obtain the ultimate stress and plastic zone width of web pillar given the mining height was 5 m:

31 $$\sigma_{1\text{max} } = 1.9536e^{{1.2x_{q} }} - 0.518$$

Based on the web pillar catastrophic instability criterion and by substituting *L*_*q*_ = 2.27*X*_*q*_ into Eq. (20), web pillar width *L*_*c*_ of 3.17 m was obtained under static loading. Therefore, the web pillar at the maximum mining depth would be under critical instability state. By substituting *L*_*c*_ into Eq. (21), the coal pillar strength *σ*_*zl*_ = 9.8249 MPa.

In this design, the permanent web pillar was not considered. Therefore, a 1.4 web pillar FoS (*f*_*s*_) was used and the maximum stress *σ*_1*max*_ was calculated to be 7.0178 MPa (Eq. 22). By substituting *σ*_1*max*_ into Eq. (23) and Eq. (24), the total width of the plastic zone was 2*X*_*q*_ = 2.26 m and the required width of the web pillar is *L*_*c*_ = 4.93 m.

### Comparison matrix

A risk assessment and hazard level system for Antaibao open cut coal mine was developed. A1, A2, A3, A4 were groups constructed according to the endpoint values of hazard level. A1, A2 are the left and right endpoints of the stable interval, A3 is the right endpoint of the mostly stable level, A4 is the right end point of the unstable level. The auger mining pillar width, depth of cover, ultimate strength, opening width, and coal pillar height were mixed into the sample group composed of empirical data for weight ranking, as shown in Table 4. Such way of integrating expert experience can enhance subjectivity, thereby balancing subjective and objective factors. This can fully reflect the importance of the indicator itself and the data information of the indicator^43–45^.

**Table 4:** Evaluation set data table.

Evaluation Indexes Sample label	Width of pillar/m	Depth of cover/m	Ultimate strength/MPa	Width of opening /m	Height of pillar/m
A1	6	50	12	1.2	1.8
A2	4	80	8.5	1.8	3.5
A3	2.5	160	4.5	3.5	5
A4	1	200	1.5	5	6.5
A5	4.93	105.5	9.825	3.3	5

Evaluation indicator data was standardised based on Eq. (29) and Eq. (30), so that the sample data are all in the [0,1] interval. By then, the cumulative transformation matrix was obtained by Eq. (26). If each value of the *m *− 1th row was greater or equal to the corresponding value in the *m*-th row, then *s*_*xy*_ = 1, otherwise record *s*_*xy*_ = 0. Subsequently, the comparison matrix *S* = (*s*_*xy*_)_*m*×*m*_ was obtained, see Table 5.

**Table 5:** Comparison matrix.

	A1	A2	A3	A4	A5
A1	1	1	1	1	1
A2	0	1	1	1	0
A3	0	0	1	1	0
A4	0	0	0	1	0
A5	0	0	1	1	1

### HASSE matrix and HASSE diagram

**Figure 6:**
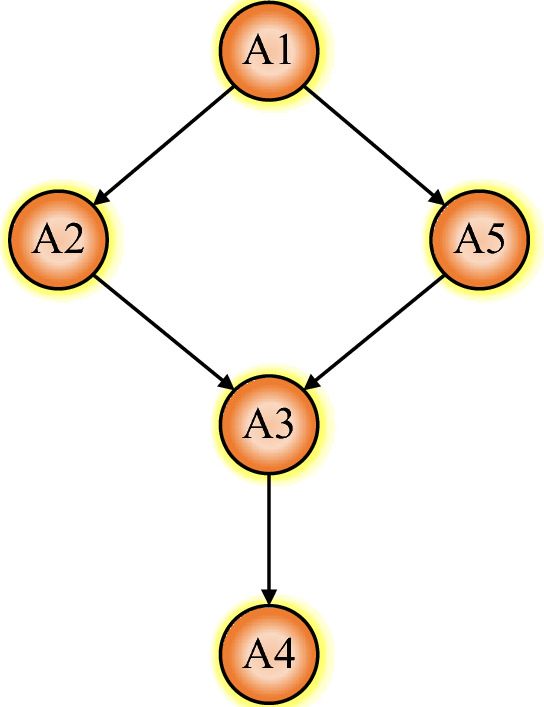
HASSE diagram.

**Figure 7:**
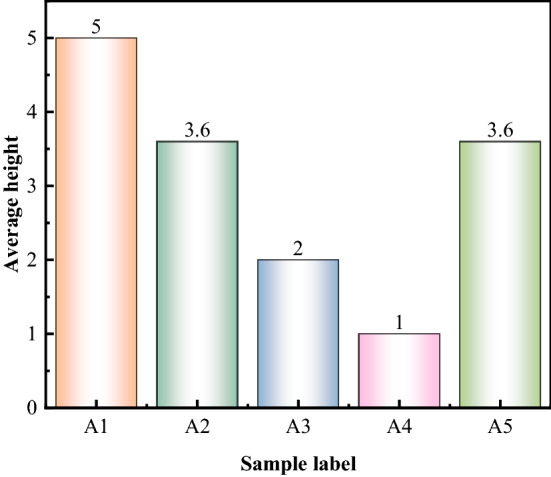
The average height of the evaluated object on the poset.

**Figure 8:**
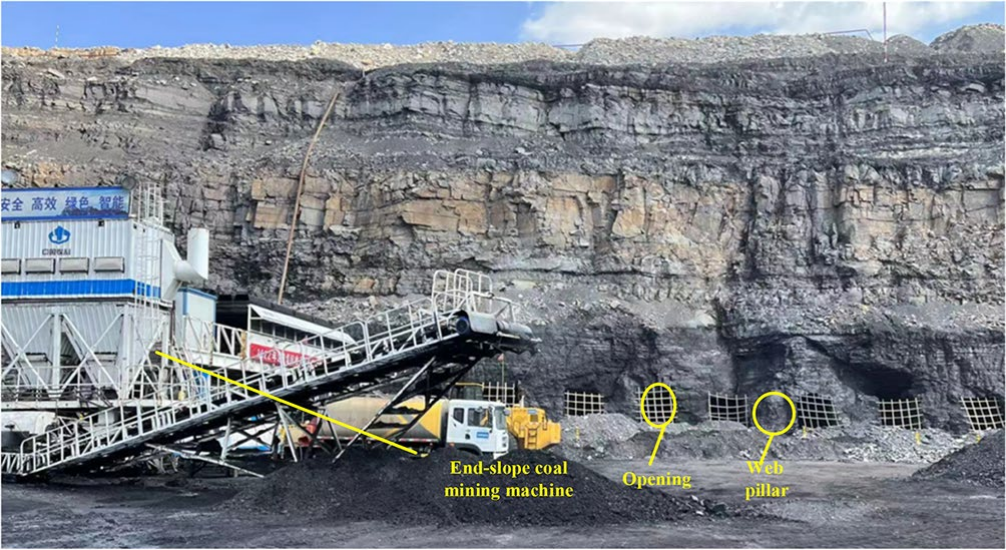
Auger mining engineering practice.

Comparison matrix can be converted to HASSE matrix by using Eq. (27), see Table 6. Based on the method proposed by Fan^37^, the HASSE diagram of poset was drawn using the HASSE matrix, as shown in Fig. 6. The HASSE diagram can visually present ranking of samples and it is divided into different layers. The samples in the same layer set have their own advantages and disadvantages, and it is difficult to distinguish between them. By comparing the heights, samples within the same layer can be sorted again. Hence the average height^46,47^ was calculated, as shown in Fig. 7. Poset ranking was developed around the notion of linear scaling^48^. For a poset, if all possible linear extensions are found, it is possible to compute the average height of individual elements and rank indicators accordingly^49^. Based on evaluation results, it was found that the average height of the evaluating object A5 was same as that of the grade sample A2, which means that the hazard levels of A5 and A2 are the same, and the coal pillar is in a mostly stable state. The field results show that the width of the web pillar was 4.93 m (Fig. 8), such that it is in a mostly stable state, Therefore, the evaluation results are consistent with the field conditions on site.

**Table 6:** HASSE matrix.

	A1	A2	A3	A4	A5
A1	0	1	0	0	1
A2	0	0	1	0	0
A3	0	0	0	1	0
A4	0	0	0	0	0
A5	0	0	1	0	0

## Conclusions

1. For web pillars under auger mining, when the ratio of plastic zone width to web pillar width is greater than 88%, the coal pillar may be unstable based on catastrophic theory. Based on limit equilibrium theory of stress–strength state in web pillar, the maximum allowable plastic yield zone width of the web pillar and minimum pillar width under different FoS were obtained.
2. The partially ordered set evaluation model was implemented to study the risk of web pillar instability in auger mining. This was able to minimise the influence of changes in index weighting on the evaluation results. The evaluation results show that pillar width was the most critical factor for pillar stability, upon completion of data normalisation. The A1, A2, A3, and A4 samples were proposed according to the endpoint values of the hazard levels, and a comparison matrix was established between the evaluating object A5 and other four clusters. Using the conversion equation, comparison matrix was converted to HASSE matrix. Based on HASSE matrix and average height from partial ordered set, it was concluded that the hazard levels of A5 and A2 are the same. The web pillars were therefore in a mostly stable state, which was consistent with the field observation.
3. Using the partial order set evaluation method can accurately determine the hazard level and state of web pillars. This can in turn improve and optimise the design of web pillars during auger mining and minimise the roof failures and landslides caused by instability of web pillars. However, current value ranges of hazard levels interval are mainly experience based, and further research and improvement are required.

## Data Availability

All data generated or analysed during this study are included in this published article.
